# Incidence and predictors of mortality among low birth weight neonates in Africa: a systematic review and meta-analysis

**DOI:** 10.3389/fped.2025.1458871

**Published:** 2025-07-15

**Authors:** Leweyehu Alemaw Mengstie, Wegayehu Zeneb Teklehaimanot, Mohammed Tesema Gebeyehu, Worku Abemie, Abatwoy Ayfokru, Yihenew Ayehu Dessie, Mitiku Tefera, Bekahegn Girma

**Affiliations:** ^1^ School of Nursing and Midwifery, Asrat Woldeyes Health Sciences Campus, Debre Berhan University, Debre Berhan, Ethiopia; ^2^ Department of Nursing, College of Health Science, Bulehora University, Bulehora, Ethiopia

**Keywords:** incidence, predictors, mortality, low birth weight, neonates, Africa, meta-analysis

## Abstract

**Background:**

In Africa, the burden of low birth weight (LBW) neonatal mortality remains substantial, yet comprehensive evidence is lacking, with varied and inconclusive findings from primary studies. This systematic review and meta-analysis aimed to assess the pooled incidence and predictors of LBW neonatal mortality in Africa.

**Methods:**

In accordance with the Preferred Reporting Items for Systematic Reviews and Meta-Analyses (PRISMA) guidelines, we systematically searched PubMed, EMBASE, Cochrane Library, African Journals Online, Web of Science, Google Scholar, and Google for relevant studies. The Newcastle Ottawa Scale was used to assess study quality. Heterogeneity and publication bias were evaluated using the *I*^2^ statistic and Egger's test. A random-effects model was applied due to significant heterogeneity, with pooled incidence and 95% confidence intervals (CIs) calculated. Subgroup and sensitivity analyses explored sources of heterogeneity.

**Results:**

This meta-analysis included 28 studies involving 56,775 LBW neonates. The pooled incidence of LBW neonatal mortality in Africa was 33.1% per 100 person-years (95% CI: 19.54–46.65), with substantial heterogeneity (*I*^2^ = 99.9%, *P* < 0.001). Predictors associated with LBW neonatal mortality included extremely low birth weight (PHR = 4.37, 95% CI: 2.62–7.29), lack of antenatal care follow-up (PHR = 2.84, 95% CI: 1.21–6.67), perinatal asphyxia (PHR = 1.73, 95% CI: 1.38–2.16), necrotizing enterocolitis (PHR = 2.80, 95% CI: 2.03–3.86), preterm birth (PHR = 3.17, 95% CI: 1.88–5.35), respiratory distress syndrome (PHR = 1.87, 95% CI: 1.57–2.23), sepsis (PHR = 2.04, 95% CI: 1.59–2.63), lack of kangaroo mother care (PHR = 5.29, 95% CI: 2.76–10.16), maternal diabetes mellitus (PHR = 2.74, 95% CI: 1.87–4.01), and maternal HIV infection (PHR = 4.47, 95% CI: 2.06–9.67).

**Conclusions:**

This study highlights a concerning high incidence of LBW neonatal mortality in Africa. Strategies targeting these predictors, such as improving antenatal care, promoting kangaroo mother care, and managing maternal health conditions like diabetes and HIV, could substantially reduce LBW neonatal mortality in the region. Policymakers and healthcare providers should prioritize these interventions to mitigate the high burden of LBW neonatal mortality and improve neonatal health outcomes across Africa.

**Systematic Review Registration:**

identifier, CRD42024560375.

## Introduction

Low birth weight (LBW), defined as birth weight of less than 2,500 g, remains a significant global public health challenge, particularly in Africa. Comprehending the Incidence and Predictors of Mortality in LBW Neonates in Africa is critical for developing effective health interventions and policies to improve neonatal outcomes (1, 2).

Low birth weight (LBW) remains a leading cause of neonatal morbidity and mortality globally, with a disproportionate burden borne by low- and middle-income countries (LMICs) (3).

According to the World Health Organisation (WHO), LBW affects approximately 15%–20% of all births worldwide, with a higher prevalence observed in low- and middle-income countries (LMICs), including those in Africa (4). Africa's distinct sociocultural and economic context marked by widespread maternal undernutrition (5), inadequate access to quality perinatal care (6), and persistent healthcare system inequities significantly contributes to the high burden of low birth weight (LBW)-related mortality (7).

Several studies have identified various predictors of mortality among LBW neonates, including prematurity, infections, birth asphyxia, and limited access to quality maternal and neonatal care (8, 9). Socioeconomic determinants such as maternal education, income level, and access to healthcare services also play crucial roles in neonatal survival (10).

Despite these findings, the incidence and predictors of mortality among low birth weight neonates differ significantly across various countries in Africa, highlighting the need for a region-specific synthesis of existing research. Therefore, this systematic review and meta-analysis aimed to determine the incidence of mortality among LBW neonates in Africa and to identify key predictors.

## Research question

What is the pooled incidence of mortality among low birth weight (LBW) neonates in Africa?

What are the main predictors associated with mortality among LBW neonates in African countries?

## Methods

### Prospero registration and reporting

This systematic review and meta-analysis has been registered in the international prospective registry of Prospero with registration number (CRD42024560375). This review has been reported in accordance with the preferred Items for Systematic Review and Meta-Analysis (PRISMA 20 statement) guideline Supplementary File 1 (11).

### Eligibility criteria

The inclusion criteria will follow the CoCoPop mnemonic (Condition, Context, and Population) since this review aims to evaluate incidence data.

**Population:** Low birth weight neonates
**Condition:** This systematic review considered studies that report on the prevalence and/or incidence, contributing factors, and outcomes of the specified condition.
**Context:** This systematic review and meta-analysis will include studies conducted in Africa.
**Types of studies:** Cohort studies from clinical and community-based settings reporting the incidence density rates of neonatal mortality and predictors using a hazard function were included.
**Language:** We considered articles published in the English language
**Publication status**: This review considered both unpublished and published articles from 2002 to 2024GC were included for analysis.

However, studies that did not report either the incidence density rate or predictors of neonatal mortality based on survival analysis principles or hazard functions were excluded. The EndNote X7 reference manager was used to organise the retrieved articles.

### Search strategy

Eligible studies were selected through a tiered process: initially by titles, followed by abstracts, and finally by full-text articles, all based on the inclusion criteria. Primary studies were identified through searches in the Cochrane Library, PubMed, CINAHL,Scopus EMBASE, HINARI, Google Scholar, and Google. Search terms were used both individually and in combination with Boolean operators, “AND” and “OR”.In addition, after the identification of included studies, cross-references were searched to identify more eligible studies.

(Incidence OR “epidemiology” OR Occurrence OR Outcome OR Magnitude OR Prevalence OR Burden OR Proportion) AND (Mortality OR Death OR Fatality rate OR Survival OR “Survival rate” OR “Time to death”) AND (Predictors OR “Associated factors” OR Determinant OR “Risk factors”) AND (“Low birth weight neonates” OR “Low birth weight infants” OR “Low birth weight new born” OR “Very low birth weight neonates” OR “Extremely low birth weight neonates” OR “Small birth weight neonates”OR “Small for gestation age” OR “Small birth weight babies” OR “Small birth weight infants” OR “Small birth weight newborns” OR “Underweight neonates” OR “Below average birth weight neonates”) AND (“Africa”) were used for searching of literature.

### Screening and data extraction

Two reviewers (LAM, WA) screened titles and abstracts against the inclusion criteria. Then, the full texts of articles were examined, by LAM, BG, YAD and MT independently. Discrepancies between reviewers were resolved through discussion.

The required data was extracted from a standard Microsoft Excel spreadsheet. For the incidence “The author's name, publication year, country, study design, sampling technique, sample size, response rate, incidence rate, and quality score were the key data types extracted. Moreover, Author name, publication year, hazard ratio (HR), lower confidence interval (LCI), and upper confidence interval (UCI) were extracted to identify predictors of low birth weight neonatal mortality.

#### Assessment of study quality

The Newcastle-Ottawa Scale (NOS) was utilized to evaluate the quality of the studies (12). LAM, WZ,AYB,MT and BG independently evaluated the quality of each included study using the appropriate appraisal tools, and any discrepancies were resolved through discussion.

the studies using this tool, considering aspects such as selection criteria, comparability, and the methodology employed for outcome determination. Studies scoring a minimum of 6 out of 10 on the Newcastle-Ottawa Scale were included in this review and meta-analysis. Although the Newcastle-Ottawa Scale (NOS) was used to assess study quality, no study was excluded based on a NOS score of less than 6.

#### Effect measures

In this review and meta-analysis, we evaluated the pooled incidence of LBW neonatal mortality, which was calculated by dividing the number of low birth weight neonates who died by the total number of person-year observations. The second objective was to assess the predictors of mortality among low birth weight neonates in Africa. In this review and meta-analysis, factors identified as determinants in at least three studies were included in meta-analysis. The pooled effect was expressed using the Hazard ratio (HR).

#### Data synthesis methods

In this study, the heterogeneity among the collected data was evaluated using the *I*^2^ test. It was then categorised into three levels: low (up to 50%), moderate (50%–75%), and high (over 75%) (13). We used STATA version 14 for analysis and a random effects model was used because of the significant variability observed among the studies. To further examine this variability and potential publication bias, we conducted the *I*^2^ test for heterogeneity and Egger's test for publication bias. The substantial heterogeneity among the studies warranted the use of a random effects model. we constructed a funnel plot to visually inspect the distribution of studies and detect potential publication bias. Subgroup and sensitivity analyses were performed to identify the sources of heterogeneity. The result of this meta-analysis was presented using figures and tables.

## Results

In this systematic review and meta-analysis, 56,775 populations were included.

### Study search and selection

**Figure 1:**
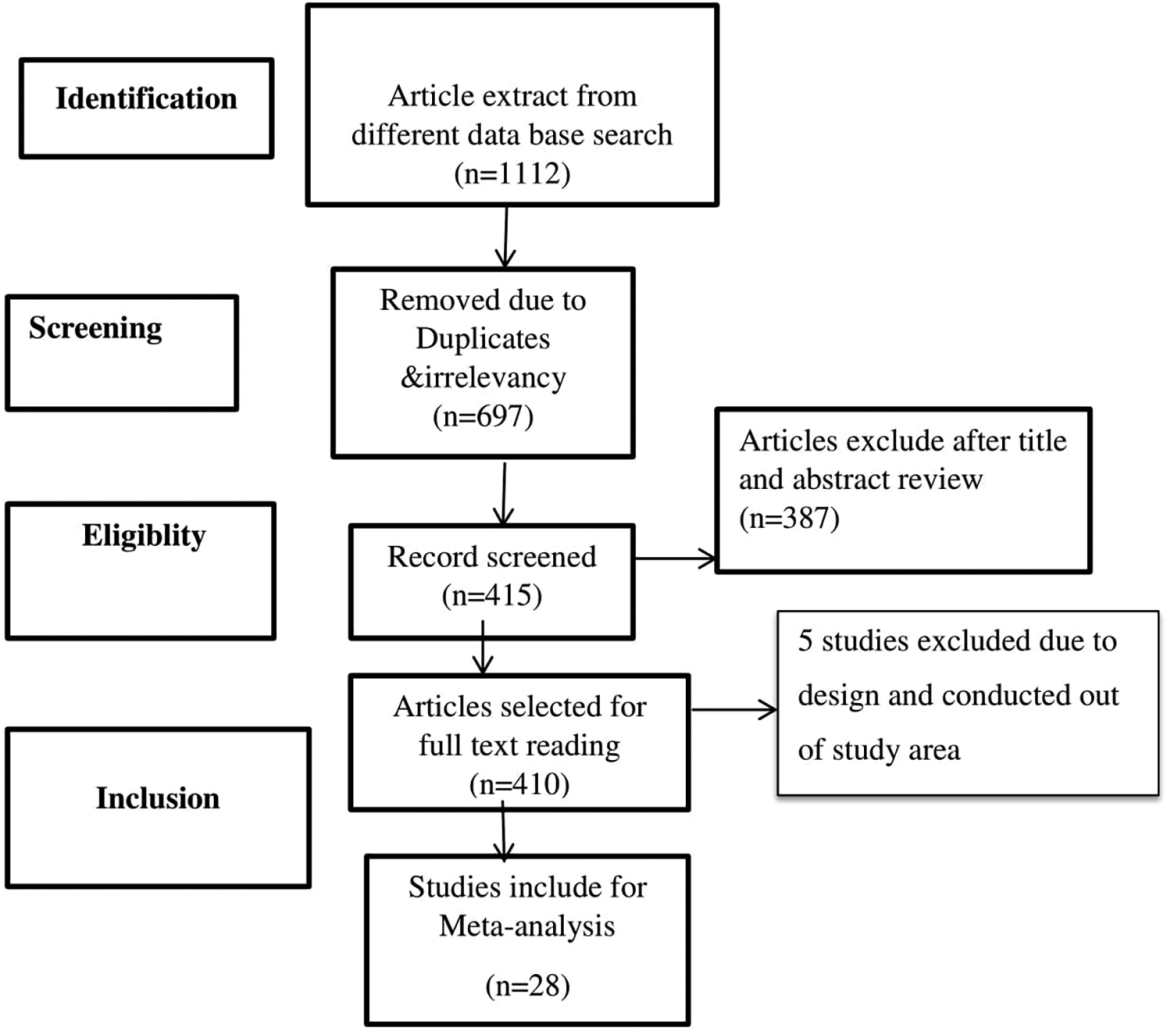
PRISMA flowchart diagram of the study selection.

We searched full-text primary studies conducted on human beings and published in the English language until now. Atotal of 1,112 primary articles were reviewed from PubMed, EMBASE, Cochrane Library, African Journal of Online, Web of Science, Google Scholar databases, and Google. Of these, 697 articles were excluded because of duplication, and 387 were excluded based on their title and abstracts.Moreover 5 studies were excluded due to different in study design and conducted out of study area. Lastly, 28 articles that met the inclusion criteria were selected for this meta-analysis (Figure 1).

### Characteristics of included studies

This systematic review and meta-analysis included 28 primary studies. The total population of the included studies was 56,775 participants. All the studies included in this review utilized a cohort study design.

From these 16 studies were a retrospective cohort. Half of the studies (14) were conducted in Ethiopia (14–22). Three of the studies were conducted in Nigeria (23–25). Two studies from Malawi (26, 27), and Another two studies were conducted in South Africa (28, 29). The rest, Zambia (30), Burkinafaso (31), Tanzania (24), Zimbabwe (32),Cameron (29, 33) and Uganda (34), with one study from each country. More than half of the included studies (15) utilized a simple random sampling technique (Table 1).

**Table 1:** Characteristics of the included studies on incidence and predictor of low birth weight neonatal mortality in Africa, 2024 (*N* = 28).

Author	Publication year	Country	Design	Sampling technique	Sample size	Included	Prev	PYO/d	Incidence	Quality Score
Birhanu et al.	2023	Ethiopia	Retrospective cohort	Simple random sampling	329	329	31.9	2608	40.25	8
Eshete et al.	2018	Ethiopia	Prospective cohort	Multistage	885	885	10.96	886	12.32	6
Kebede et al.	2022	Ethiopia	Retrospective cohort	Simple random sampling	384	358	35.2	3,328	37.86	7
Coulibaly et al.	2016	Burkinafaso	Prospective cohort	Multistage	341	341	5.2	3,358	53	7
Wondie et al.	2023	Ethiopia	Retrospective cohort	Simple random sampling	793	761	32.46	3,266	75.63	8
Dessu et al.	2020	Ethiopia	Prospective cohort	Simple random sampling	216	216	8.3	1,240	14.5	7
Debere et al.	2022	Ethiopia	Prospective cohort	Cluster sampling	1,092	808	29.95	1,496	16.17	8
wolelie et al.	2020	Ethiopia	Retrospective cohort	Simple random sampling	718	718	28.1	5,715	35.3	8
Nduna et al.	2015	zambia	Prospective cohort	Purpusive sampling	148	148	30	1,493	29.7	7
Um SS et al.	2020	Camerun	Retrospective cohort	Simple random sampling	10,590	10,590	9.1	10,582	91	7
Gebrekidan et al.	2024	Ethiopia	Retrospective cohort	Simple random sampling	329	329	21.56	2,346	30.26	8
Njim et al.	2015	Camerun	Retrospective cohort	Cluster sampling	4,941	4,941	1.9	4,933	19.03	7
Onyiriuka et al.	2010	Nigeria	Prospective cohort	Systematic random sampling	191	191	21.6	4,177	51.7	7
Zeleke et al.	2012	Ethiopia	Prospective cohort	Systematic random sampling	309	305	56.1	3,280	17.1	7
Nsubuga et al.	2024	Uganda	Prospective cohort	Systematic random sampling	220	216	6.5	2,166	6.48	7
Ballot et al.	2010	Southa frica	Retrospective cohort	Simple random sampling	488	474	29.5	4,745	29.5	7
Genie et al.	2021	Ethiopia	Retrospective cohort	Simple random sampling	319	291	37.8	2,915	37.7	8
Mengstie et al.	2022	Ethiopia	Retrospective cohort	Simple random sampling	416	416	25.7	2,498	42.83	8
Abraham et al.	2018	Ethiopia	Retrospective cohort	Systematic random sampling	193	161	23	1,611	22.98	7
Tesema et al.	2023	Ethiopia	Retrospective cohort	Simple random sampling	300	300	28.3	2,437	34.9	7
Nigussie et al.	2024	Ethiopia	Prospective cohort	Multistage	768	768	28.3	5,599	38.8	8
Mashingo et al.	2019	Malawi	Retrospective cohort	Systematic random sampling	185	185	23	1,896	23.2	7
Mvunta et al.	2019	Tanzania	Prospective cohort	Multistage	26,191	26,191	28.1	26,191	28.1	8
Zgambo et al.	2021	Malawi	Retrospective cohort	Purpusive sampling	1,343	1,343	13	7,309	23	8
Osuorah et al.	2019	Nigeria	Retrospective cohort	Simple random sampling	166	166	31.8	1,661	31.9	8
Chukuwundi et al.	2002	Nigeria	Retrospective cohort	Simple random sampling	280	280	1.43	3,160	12.64	7
Crichton et al.	2017	South Africa	Prospective cohort	Simple random sampling	4,665	4,665	19.8	4,667	19.8	8
Zvenyika et al.	2018	Zimbabwe	Prospective cohort	Simple random sampling	399	367	51.2	367	51.2	7

N.b: PYO/d: person year observation in days.

#### Results of syntheses and reporting bias

**Figure 2:**
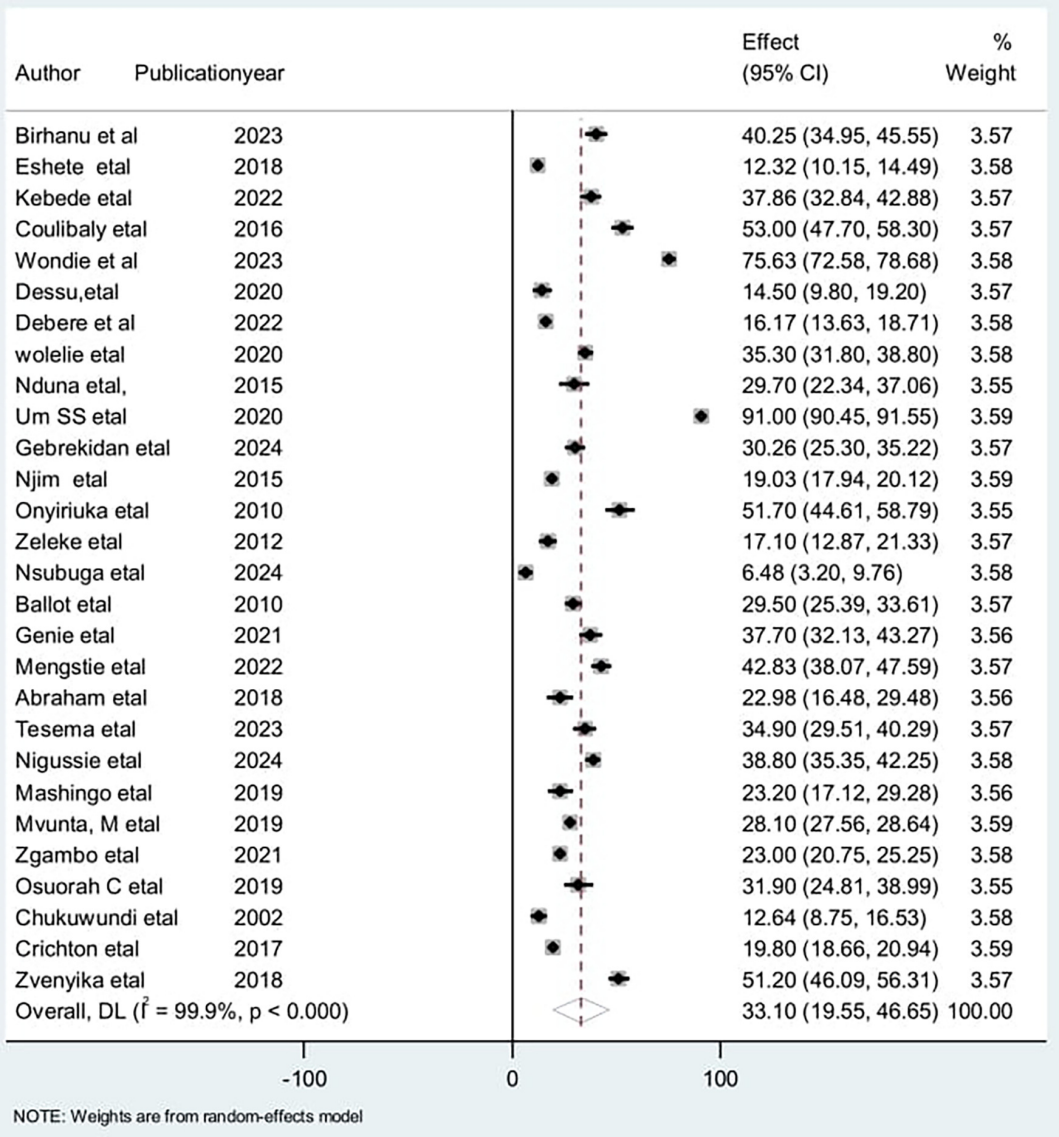
Forest plot for the incidence of low birth weight neonatal mortality in Africa, 2024 (*n* = 28).

A forest plot was done to display the outcomes of the included 1 studies. This systematic review and meta-analysis comprised 28 primary studies to estimate the pooled incidence of mortality among low birth weight (LBW) neonates. In the present systematic review and meta-analysis, the pooled incidence of mortality among low birth weight neonates in Africa was 33.1%, (95% CI: 19.54–46.65) per 100 per year observation (Figure 2).

**Figure 3:**
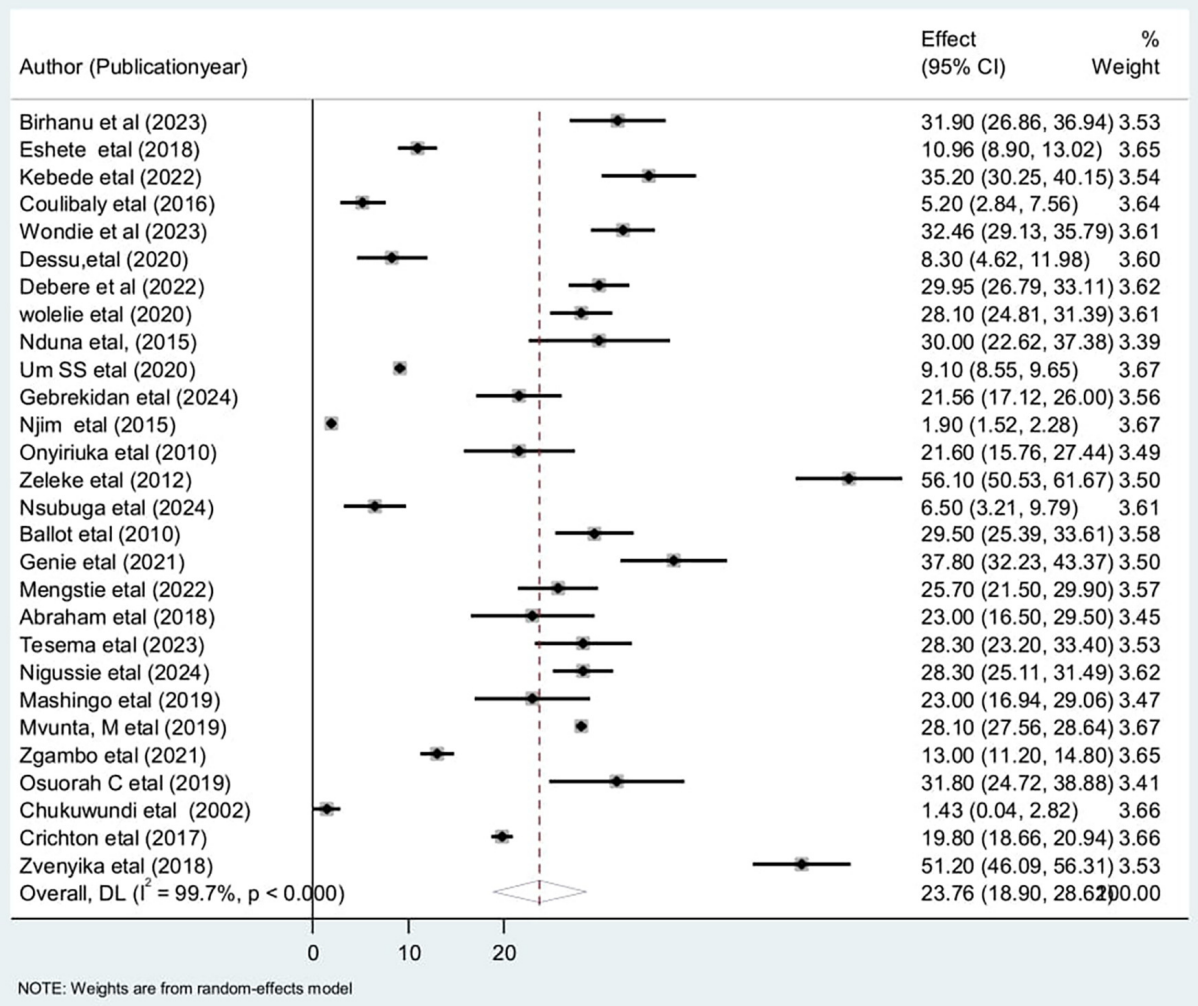
Forest plot for the overall prevalence of low birth weight neonatal mortality.

A total of 23.75% (95% CI: 18.89, 28.62) of the participants died during the follow-up period (Figure 3).

**Figure 4:**
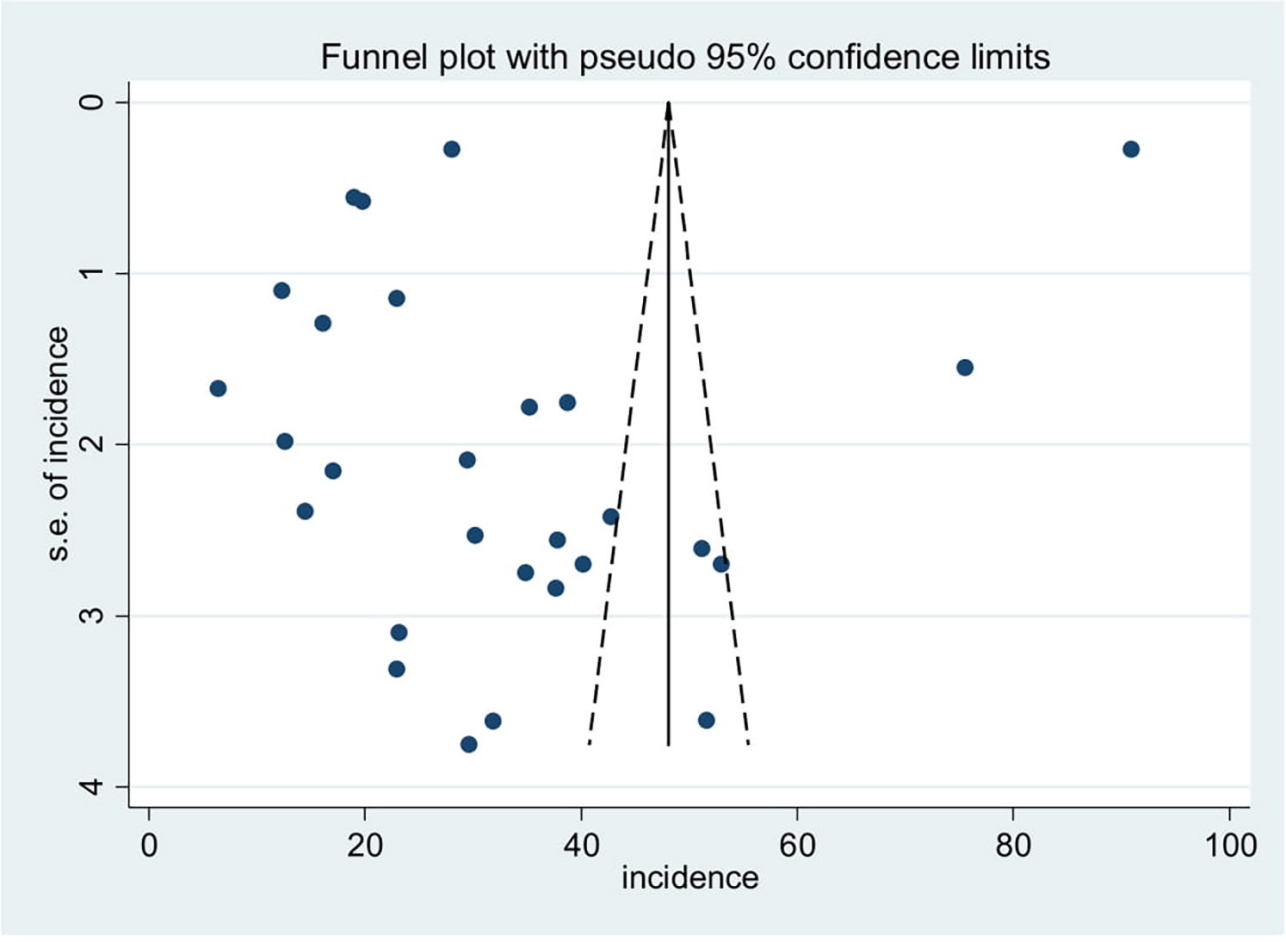
Funnel plot for the incidence of low birth weight neonatal mortality and its predictor in Africa.

The presence of heterogeneity and publication bias was evaluated within the included studies. There was significant heterogeneity across the studies in this meta-analysis (*I*^2^ = 99.9%, *p* < 0.001). There was no publication bias among the included studies, even though the funnel plot shows an asymmetrical distribution (Figure 4). However, Egger's test did not indicate a statistically significant presence of publication bias (*p* = 0.143).

**Figure 5:**
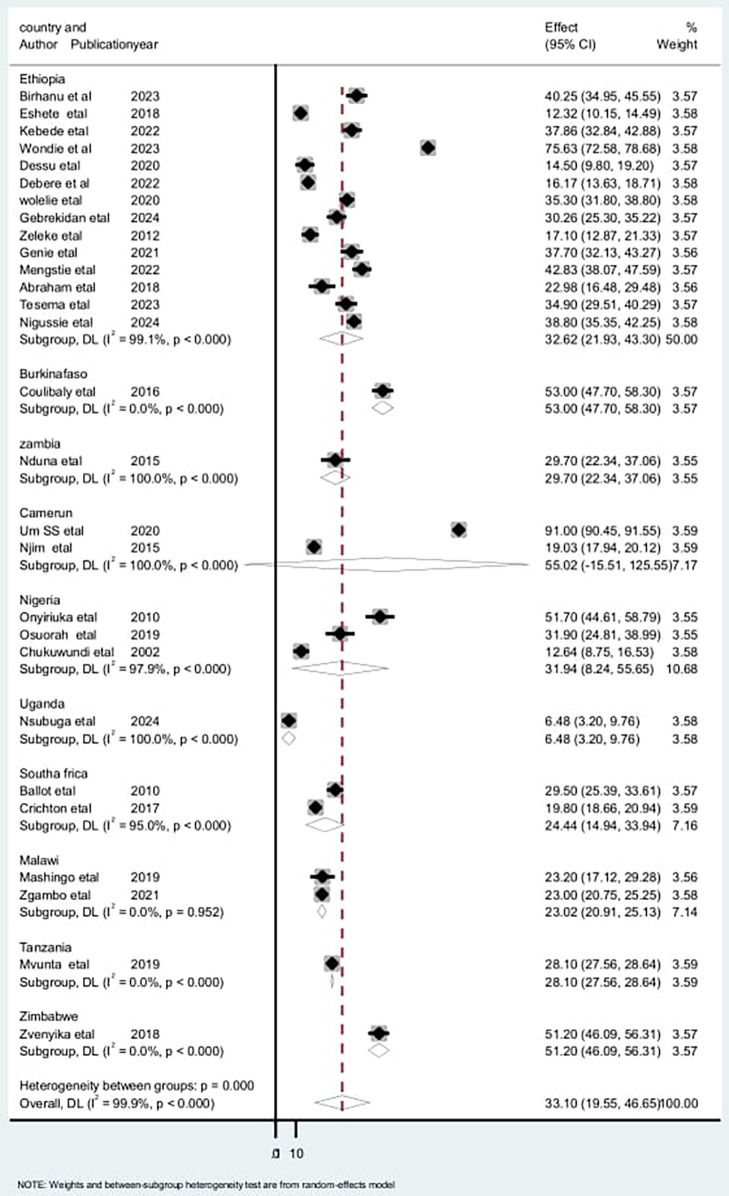
Subgroup analysis by countries for the included studies to investigate the source of heterogeneity among studies conducted in Africa, 2024 (*n* = 28).

**Figure 6:**
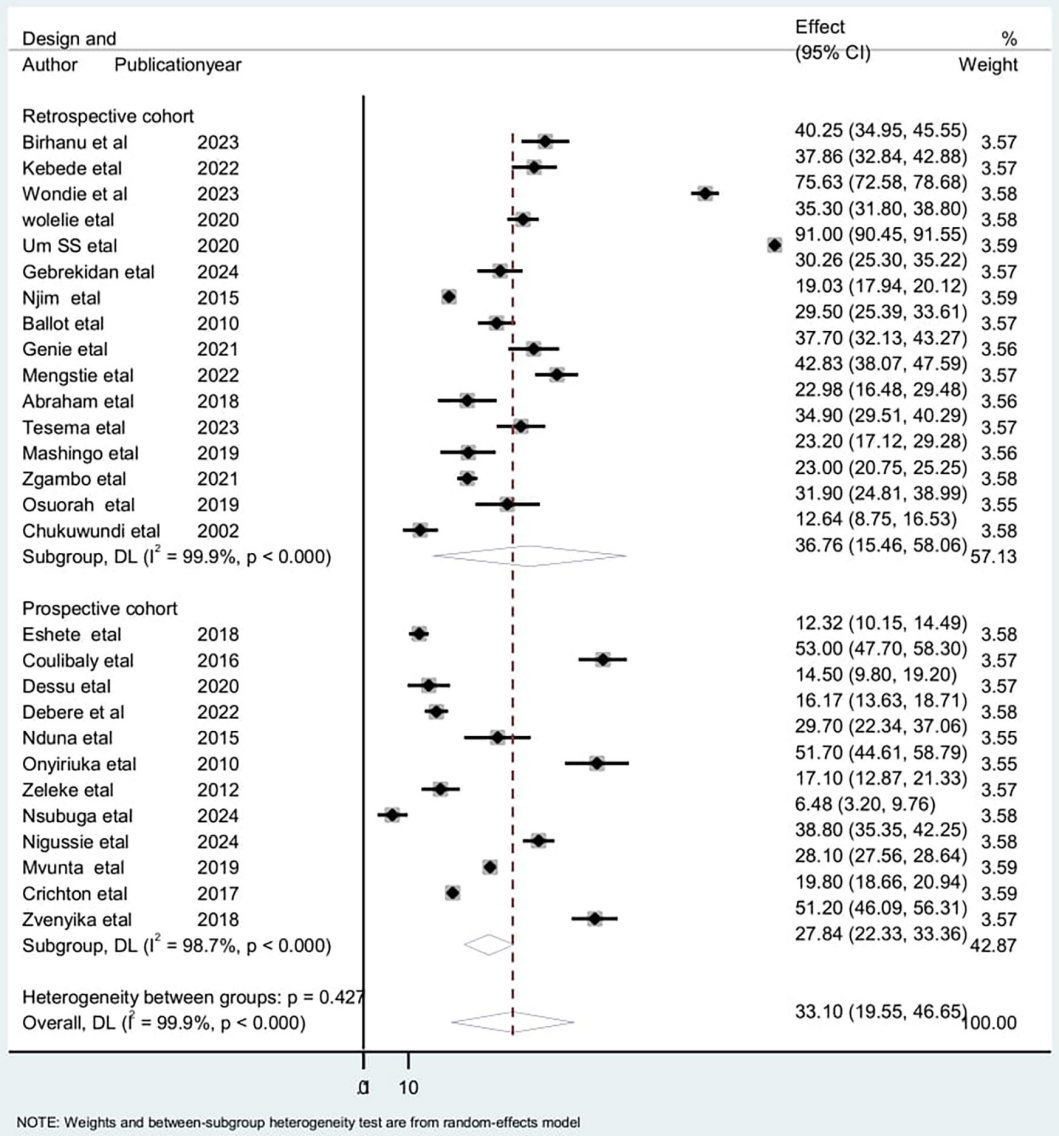
Sub group analysis by study design for the included studies to investigate the source of heterogeneity among studies conducted in Africa, 2024 (*n* = 28).

**Figure 7:**
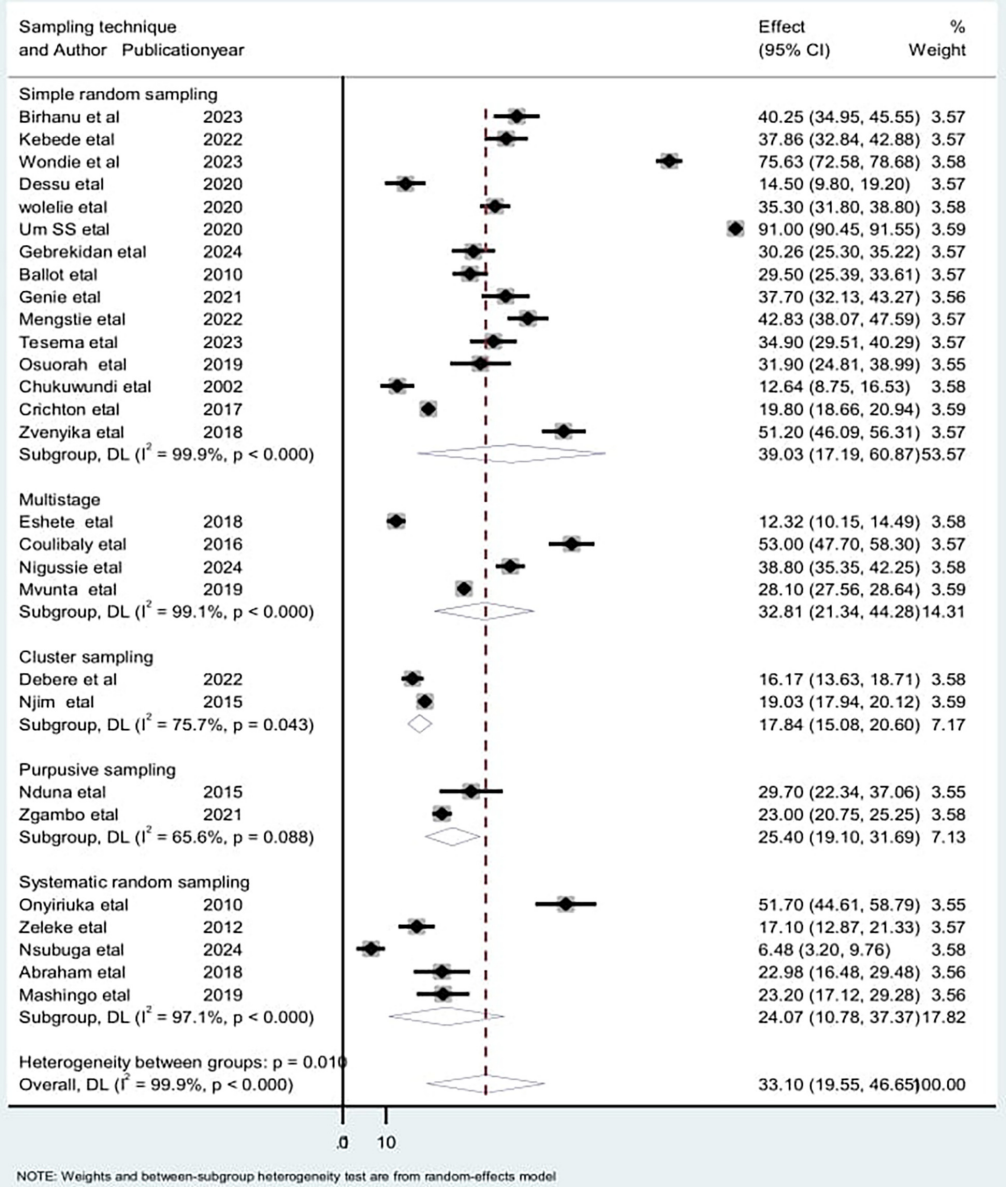
Sub group analysis by sampling technique for the included studies to investigate the source of heterogeneity among studies conducted in Africa, 2024 (*n* = 28).

**Figure 8:**
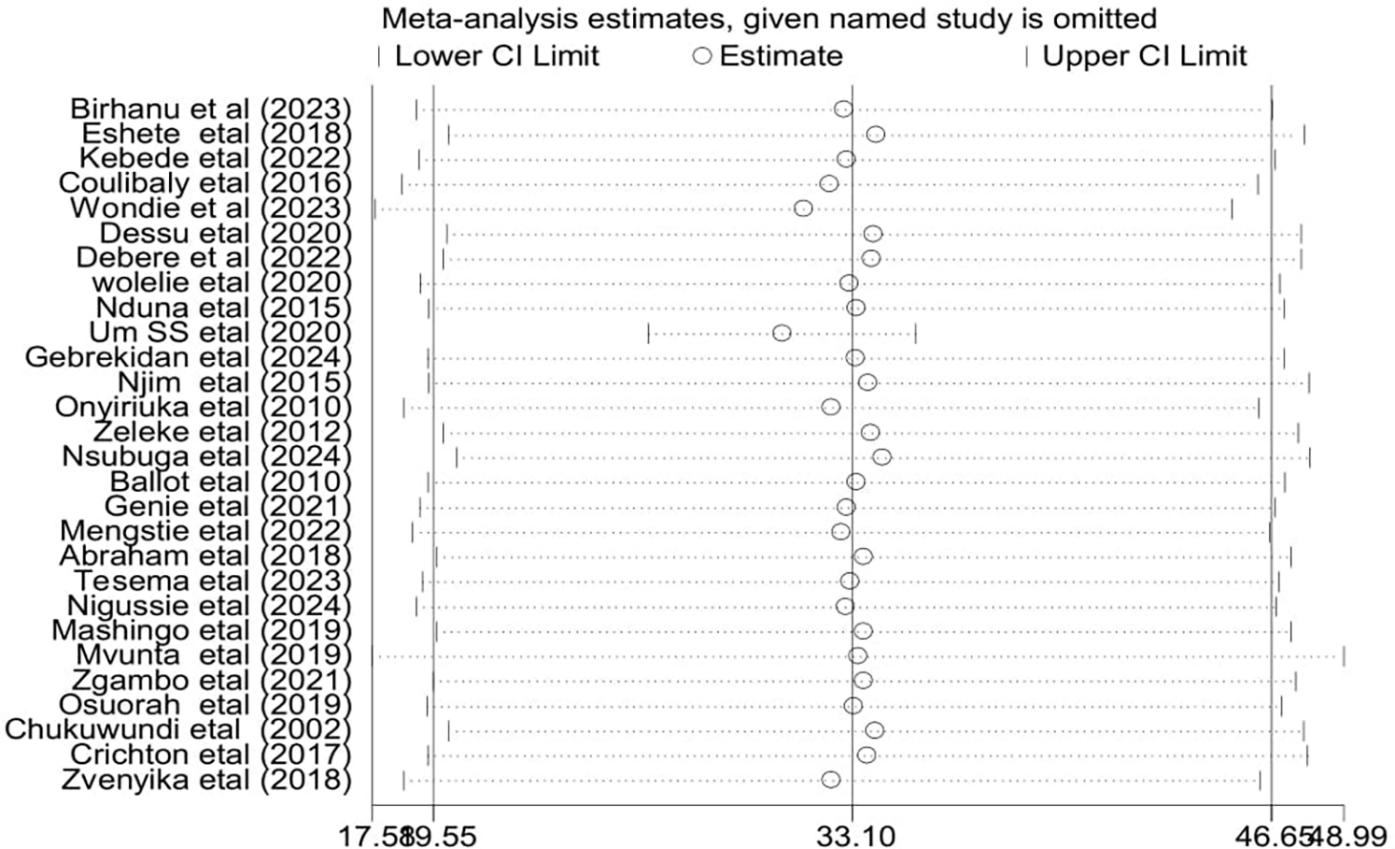
Sensitivity analysis for studies included to estimate the pooled incidence of low birth weight neonatal mortality in Africa.

To assess the source of heterogeneity, a subgroup analysis was conducted based on the study area, study design, and sampling technique. Studies conducted in Cameroon had a high incidence of low birth weight neonatal mortality [55.1: 95% CI: (15.51–125.54)] and heterogeneity (*I*^2^ = 100% with *p* < 0.01), compared to studies conducted in other countries (Figure 5). Regarding the subgroup analysis on sampling design, there was a high incidence among studies on retrospective cohorts [36.76; 95% CI: (15.46–58.06)] and high heterogeneity (*I*^2^ = 99.9% with *p* < 0.001) (Figure 6). Additionally, there was a high incidence of mortality among studies with a simple random sampling technique [39.1: 95% CI: (17.19–60.87)] and high heterogeneity (*I*^2^ = 99.9% with *p* < 0.001) (Figure 7). Finally, a sensitivity analysis was conducted and revealed that no single study affect the pooled incidence rate (Figure 8).

### Predictor of mortality

A total of 28 studies were analyzed to estimate the pooled hazard ratio for predictors of mortality among low birth weight neonates.

In this study, LBW neonates with extremely very low birth weight (EVLBW) had a 4.3-fold higher risk of mortality compared to their counterparts (PHR = 4.37; 95% CI: 2.62–7.29) (14–16, 19, 35, 36). LBW neonates with perinatal asphyxia (PNA) had a 1.73 fold higher risk of mortality compared to neonates without PNA. (PHR = 1.73, 95%: CI: 1.38–2.16) (14, 16, 21, 26). Similarly, LBW neonates with respiratory distress syndrome (RDS) had a 1.87-fold higher risk of mortality (PHR = 1.87, 95% CI: 1.57, 2.23) (16, 16, 18, 26, 36, 37).

LBW neonates whose mothers had no antenatal care (ANC) follow-up had a 2.84-fold higher risk of mortality compared to their counterparts. (PHR = 2.84, 95% CI: 1.21, 6.67) (15, 22, 24). Similarly, LBW neonates with necrotizing enterocolitis (NEC) had a 2.8 fold higher risk of mortality compared to their counterparts (PHR = 2.80, 95% CI: 12.03, 3.86) (16, 16, 18). Moreover, LBW neonates born preterm had a 3.1-fold higher risk of mortality compared to those born at term (PHR = 3.1; 95% CI: 1.88–5.35) (14–16, 19, 21, 36, 38, 39). The likelihood of LBW neonates with sepsis had a 2.04 time higher risk of death (PHR = 2.04, 95% CI: 1.59, 2.63).

This review also demonstrated that LBW neonates whose mothers did not receive kangaroo mother care (KMC) were at a 5.29 times higher risk of mortality compared to their counterparts (PHR = 5.29, 95% CI: 2.76, 10.16) (14, 17, 40).

Low-birth weight neonates born to mothers with diabetes mellitus (DM) had a 2.7 times higher risk of mortality compared to their counterparts (PHR = 1.87, 4.01, 95%) (17, 36).

LBW neonates from mothers with HIV were 4.4 times more hazardous to die than mothers without HIV (PHR: 4.47, 95% CI: 2.06, 9.67) (17, 22, 24, 36) (Table 2).

**Table 2:** Meta-analysis of predictor of low birth weight neonatal mortality, 2024.

Variables	Categories	Included studies	Pooled HR (95% CI)	*Q*-statics	*P*-value of *Q*	*I*^2^ (%)	Tau^2^	*P*-value of estimates
Low birth weight	EVLBW VLBW	9 Ref	4.37 (2.62, 7.29)	44.98	<0.001	82.2	0.48	<0.001
ANC follow up	No Yes	3 Ref	2.84 (1.21, 6.67)	17.07	<001	88.3	0.47	0.016
PNA	Yes No	4 Ref	1.73 (1.38, 2.16)	3.06	0.383	1.9	0.01	<0.001
NEC	Yes No	3 Ref	2.80 (2.03, 3.86)	0.21	0.89	0	<001	<0.001
Preterm	Yes No	7 Ref	3.17 (1.88, 5.35)	28.43	<0.001	78.9	0.30	<0.001
RDS	Yes No	6 No	1.87 (1.57, 2.23	3.17	0.67	0	<0.001	<0.001
Sepsis	Yes No	4 Ref	2.04 (1.59, 2.63)	5.79	0.122	48.2	0.031	<0.001
KMC	No Yes	4 Ref	5.29 (2.76, 10.16)	2.95	0.40	0	<0.001	<0.01
Maternal DM	Yes No	3 Ref	2.74 (1.87, 4.01)	1.37	0.50	0	<0.001	<0.001
Maternal Hiv infection	Yes No	4 Ref	4.47 (2.06, 9.67)	11.49	0.009	73	0.41	<0.001

ELBW, extremely birth weight; VLBW, very low birth weight; NEC, necrotizing enterocolitis; RDS, respiratory distress syndrome; NEC, necrotizing enterocolitis; RDS, respiratory distress syndrome; KMC, kangaroo mother care; PHR, pooled Hazard ratio; DM, diabetic mellitus.

## Discussion

The pooled mortality rate among low birth weight (LBW) neonates in Africa was 33.1% (95% CI: 19.54–46.65) per 100 person-years. This result aligns with studies from Ethiopia, where the neonatal mortality rate for LBW infants has been reported to be approximately 30% (41), and in Nigeria, the neonatal mortality rate for LBW 35%–40% (42). This consistency suggests that certain regions within Africa share similar healthcare challenges and resource limitations.

This review and meta-analysis found higher mortality rates in Africa compared to high-income countries. In the United States, the rate is 5%–10% per 100 person-years (43), Sweden, it is about 4% (44), in South Africa, the neonatal mortality rate for LBW infants is about 20% (45). In Egypt, the rate is around 25% (46). This disparity is might be because of the advanced healthcare system differences, including the availability of neonatal intensive care units (NICUs) and universal healthcare access,and comprehensive prenatal and postnatal care, including routine screenings and early interventions, in high-income countries.

Healthcare system differences, including the availability of neonatal intensive care units (NICUs) and universal healthcare access.However, this finding is less than a study conducted in Bangladesh, which reported a rate of 11.2 per 100 neonate days (95% CI: 9.1–136.) (47). The variation might be due to differences in healthcare infrastructure, socioeconomic conditions, and possibly more effective maternal and child health programs.

This review identified various factors associated with mortality among low birth weight neonates. Neonates born with extremely low birth weight were found to face a four fold higher risk of mortality compared to those born with normal birth weight.

This finding in line with research conducted in Brazil (48), in England (49), India (50), Ethiopia (18) Ethiopia (15), Pakistan (51), Zimbabwe (32). This may occur because lower birth weight makes neonates more vulnerable to infections, hypothermia, and hypoglycaemia, increasing their risk of mortality.

Additionally, low birth weight (LBW) neonates with perinatal asphyxia (PNA) had nearly twice the risk of mortality compared to those without PNA. This observation aligns with findings from research conducted in Brazil (52), India (50), and Bangladish (53). This might be due to attributed to perinatal asphyxia (PNA), which results in oxygen deprivation, leading to progressive hypoxemia and hypercapnia. This condition can lead to harm in the central nervous system and other organs. In this review and meta-analysis, neonates diagnosed with Respiratory Distress Syndrome (RDS) were nearly two times higher risk of death as compared to their counterparts. This result is consistent with findings in Brazil (54), Zimbabwe (32), India (50, 55), and Ethiopia (18). Due to a significant number of neonates being born prematurely, their lungs may experience inadequate surfactant production, leading to frequent instances of collapsed lungs and respiratory failure.

Moreover, in this review, the risk of mortality among low-birth-weight newborns with NEC was nearly three times as high as that of their counterparts.This finding is consistent with a study conducted in, south Africa (28), and Brazil (54). This study shows that most subjects were preterm, making them susceptible to gastrointestinal immaturity in motility, digestive function, circulatory regulation, barrier protection, and immune defense, which increases the risk of death.

In this review, preterm low-birth-weight (LBW) neonates had a higher risk of death compared to their counterparts. This finding is consistent with studies conducted in Bangladesh (56), Burkina Faso (57), and Cuiaba (58). This is because premature neonates have an immature immune system, less adipose tissue, and incomplete organ development, making them more susceptible to complications that can lead to death.

The study found that low-birth-weight (LBW) neonates diagnosed with sepsis had a higher mortality rate compared to their counterparts. This result is supported by studies conducted in Odisha, India (58), Bahirdar (18, 57–59), and Northern India (18, 58–61). This could be due to the fact that sepsis markedly raises the likelihood of mortality. Low-birth-weight (LBW) neonates born to mothers with a history of HIV had more than four times higher risk of death compared to those born to HIV-negative mothers. This finding aligns with a study conducted in Southern Ethiopia (61). This increased risk may be due to maternal immune compromise, which limits breastfeeding and increases medical costs.

Neonates with low birth weight born from mothers with a history of diabetes mellitus had nearly three times higher risk of mortality compared to those who had not. This finding is consistent with a study conducted in southern Ethiopia (15, 17). The increased risk may be related to complications of diabetes mellitus during pregnancy, such as hypoglycaemia, hypocalcemia, respiratory distress, growth restriction, polycythemia, elevated magnesium levels, congenital anomalies, and increased bilirubin levels (62, 63).

This review revealed that neonates with low birth weight who were not placed under kangaroo mother care within one hour of delivery had five times higher risk of mortality compared to their counterparts (17). This might be because of kangaroo mother care, which facilitates early and uninterrupted skin-to-skin contact between mother and baby, and encourages exclusive breastfeeding as recommended by the WHO (64).

LBW neonates whose mothers did not receive antenatal care (ANC) follow-up were almost three times more likely to die compared to those whose mothers received ANC. This result corresponds with findings from a study conducted in Brazil (65). This might be dueto mothers who receive regular antenatal care (ANC) are more likely to detect and manage potential health issues during pregnancy.

### Limitation of the study

Most included studies were from a limited number of African countries (e.g., Ethiopia and Nigeria), which may affect the generalizability of the findings to the entire continent. Additionally, only studies published in English were included, which may have excluded relevant data. Furthermore, there was high heterogeneity among the included studies.

## Conclusions and recommendations

This meta-analysis indicates a higher incidence of mortality among low birth weight neonates in Africa. Extremely low birth weight, lack of antenatal care, perinatal asphyxia, necrotizing enter colitis, preterm birth, respiratory distress syndrome, sepsis, lack of kangaroo mother care, maternal diabetes mellitus, and maternal HIV infection were predictors of LBW neonatal mortality.

Based on these findings, it is recommended to increasing ANC visit adherence per WHO guidelines. Additionally, healthcare protocols should prioritize early detection and intervention for conditions for perinatal asphyxia, necrotizing enterocolitis, and respiratory distress syndrome. Promoting kangaroo mother care practices is also crucial for improving outcomes of low birth weight neonates. Furthermore, systematic screening and effective management of maternal diabetes mellitus and HIV infection are essential to minimize neonatal mortality. Integrating these strategies into maternal and neonatal healthcare policies can effectively mitigate the risk factors associated with low birth weight neonatal mortality.

Moreover, this study highlights key predictors of LBW neonatal mortality, though heterogeneity limits the generalizability of findings. Future research should prioritize context-specific interventions, with a focus on targeted strategies like improving neonatal resuscitation training and expanding preterm birth prevention efforts.

## Data Availability

The original contributions presented in the study are included in the article/Supplementary Material, further inquiries can be directed to the corresponding author.
